# Crystal structure of 2-[(1*R*,2*R*,4a*S*,8a*S*)-2-hy­droxy-2,5,5,8a-tetra­methyl­deca­hydro­naphthalen-1-yl]-*N*-(*o*-tol­yl)acetamide

**DOI:** 10.1107/S2056989015017600

**Published:** 2015-09-26

**Authors:** Dang-Dang Li, Xin-Wei Shi, Qiang-Qiang Lu, Sheng-Kun Li

**Affiliations:** a Lab. for Pesticide Synthesis, Department of Pesticide Science, College of Plant Protection, Nanjing Agricultural University, Weigang 1, Xuanwu District, Nanjing 210095, People’s Republic of China; b Xi’an Botanical Garden, Institute of Botany of Shaanxi Province, Xi’an 710061, People’s Republic of China

**Keywords:** crystal structure, sclareolide, sclareol, hydrogen bonding, C—H⋯π inter­actions

## Abstract

The title compound, C_23_H_35_NO_2_, is an amide derivative of the lactone (+)-sclareolide, and was synthesized from natural sclareol. In the mol­ecular structure, the two six-membered rings (*A* and *B*) of the labdane skeleton are *trans*-fused, and adopt chair conformations. There is an intra­molecular N—H⋯O hydrogen bond present forming an *S*(7) ring motif. In the crystal, O—H⋯O hydrogen bonds link the mol­ecules into helical chains propagating along the *b-*axis direction. The chains are linked *via* C—H⋯π inter­actions, forming a three-dimensional structure.

## Related literature   

For the chemistry and biological importance of sclareol and sclareolide, see: Barrero *et al.* (2004 ▸); Huang *et al.* (2001 ▸); Mohamad *et al.* (2005 ▸); Sy & Brown (1997 ▸). For the synthesis of coronarin and chinensines, see: Margaros & Vassilikogiannakis (2007 ▸). For related structures, see: Bernardinelli & Giersch (1985 ▸); Shi *et al.* (2015 ▸).
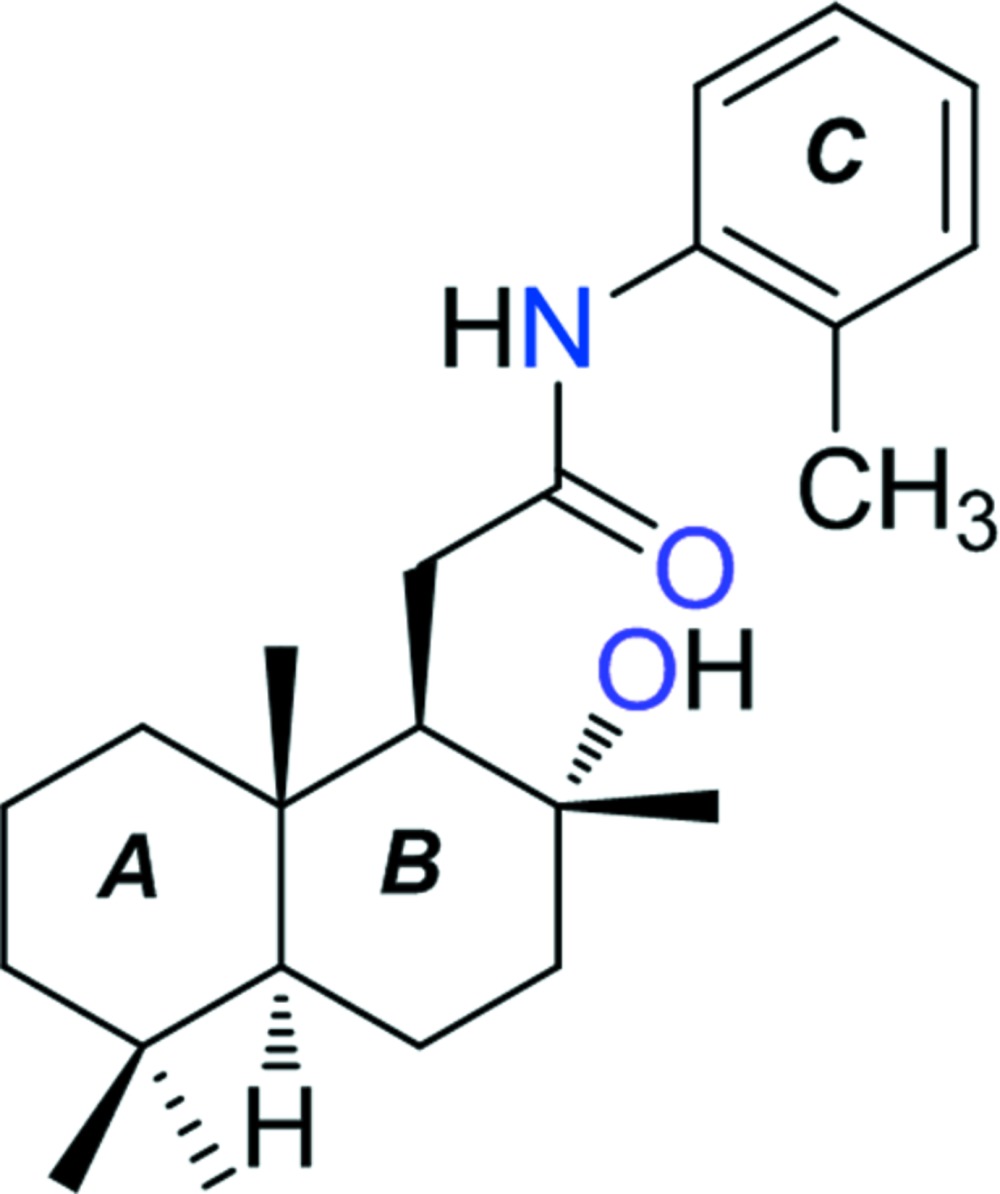


## Experimental   

### Crystal data  

C_23_H_35_NO_2_
*M* *_r_* = 357.52
Monoclinic, 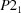

*a* = 6.3001 (5) Å
*b* = 13.2663 (10) Å
*c* = 12.7082 (10) Å
β = 96.983 (2)°
*V* = 1054.26 (14) Å^3^
*Z* = 2
Mo *K*α radiation
μ = 0.07 mm^−1^
*T* = 296 K
0.22 × 0.20 × 0.18 mm

### Data collection  

Bruker SMART APEX CCD diffractometer
Absorption correction: multi-scan (*SADABS*; Bruker, 2002 ▸) *T*_min_ = 0.985, *T*_max_ = 0.987
3714 measured reflections
3714 independent reflections
3121 reflections with *I* > 2σ(*I*)
*R*_int_ = 0.020

### Refinement  

*R*[*F* ^2^ > 2σ(*F* ^2^)] = 0.040
*wR*(*F* ^2^) = 0.095
*S* = 1.06
3714 reflections
242 parameters
1 restraint
H-atom parameters constrained
Δρ_max_ = 0.11 e Å^−3^
Δρ_min_ = −0.13 e Å^−3^

### 

Data collection: *SMART* (Bruker, 2002 ▸); cell refinement: *SAINT* (Bruker, 2002 ▸); data reduction: *SAINT*; program(s) used to solve structure: *SHELXS97* (Sheldrick, 2008 ▸); program(s) used to refine structure: *SHELXL97* (Sheldrick, 2008 ▸); molecular graphics: *SHELXTL* (Sheldrick, 2008 ▸); software used to prepare material for publication: *SHELXTL*).

## Supplementary Material

Crystal structure: contains datablock(s) I, New_Global_Publ_Block. DOI: 10.1107/S2056989015017600/su5209sup1.cif

Structure factors: contains datablock(s) I. DOI: 10.1107/S2056989015017600/su5209Isup2.hkl

Supporting information file. DOI: 10.1107/S2056989015017600/su5209Isup3.cdx

**Fig. 1.**
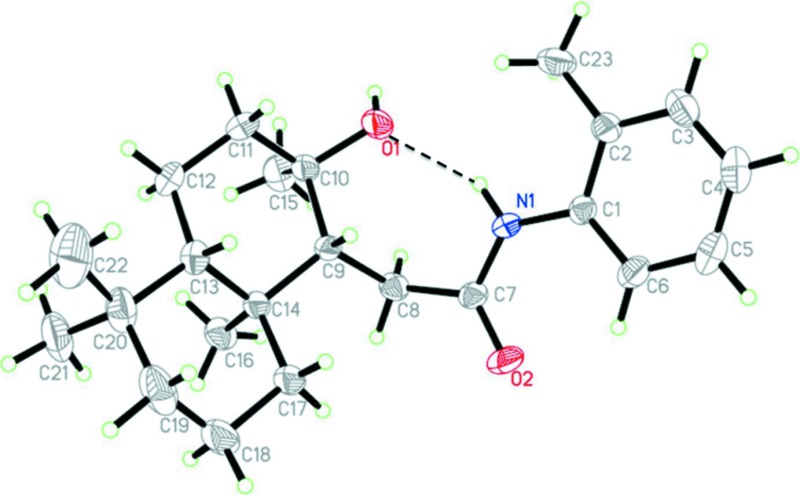
A view of the molecular structure of the title compound, with atom labelling. Displacement ellipsoids are drawn at the 30% probability level. The intramolecular N—H···O hydrogen bonds is shown as a dashed line (see Table 1).

**Fig. 2.**
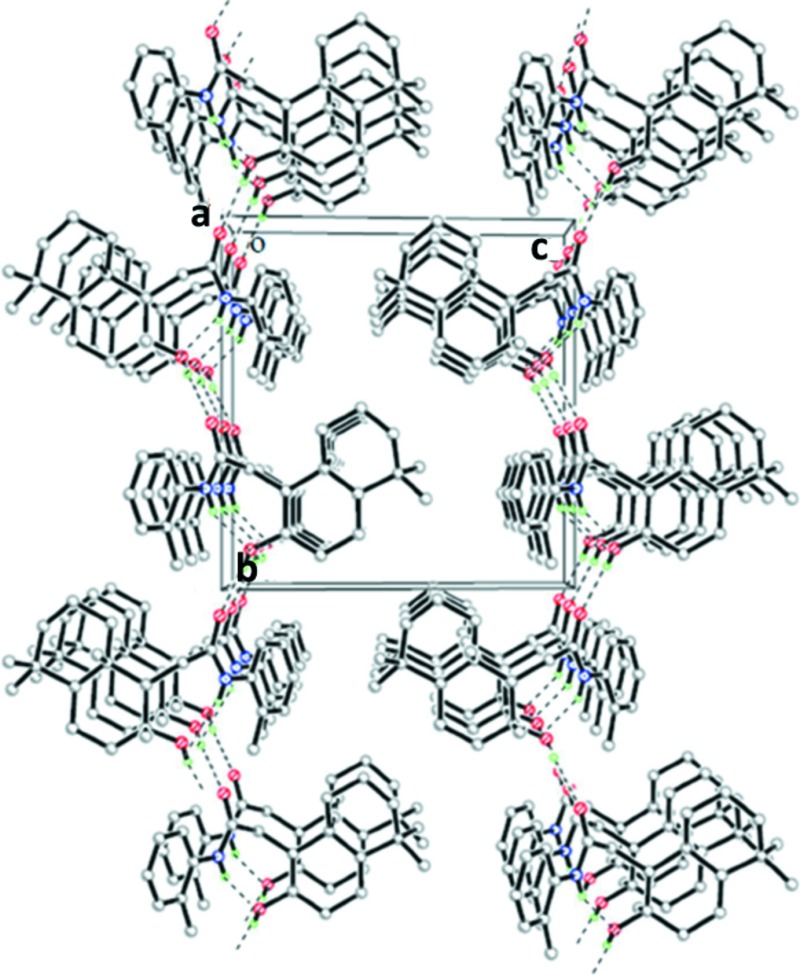
Crystal packing of the title compound, viewed along the a axis. The hydrogen bonds are shown as dashed lines (see Table 1), and C-bound H atoms have been omitted for clarity.

. DOI: 10.1107/S2056989015017600/su5209fig1.tif
A view of the mol­ecular structure of the title compound, with atom labelling. Displacement ellipsoids are drawn at the 30% probability level. The intra­molecular N—H⋯O hydrogen bonds is shown as a dashed line (see Table 1).

a . DOI: 10.1107/S2056989015017600/su5209fig2.tif
Crystal packing of the title compound, viewed along the *a* axis. The hydrogen bonds are shown as dashed lines (see Table 1), and C-bound H atoms have been omitted for clarity.

CCDC reference: 1426216

Additional supporting information:  crystallographic information; 3D view; checkCIF report

## Figures and Tables

**Table 1:** Hydrogen-bond geometry (, ) *Cg* is the centroid of benzene ring C1C6.

*D*H*A*	*D*H	H*A*	*D* *A*	*D*H*A*
N1H1O1	0.86	2.09	2.894(2)	155
O1H1*O*O2^i^	0.82	2.00	2.8054(19)	168
C8H8*B* *Cg* ^ii^	0.97	2.79	3.632(2)	146
C22H22*A* *Cg* ^iii^	0.96	2.98	3.808(3)	145

Symmetry codes: (i) 
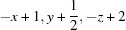
; (ii) 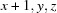; (iii) 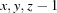.

## supplementary crystallographic information

### S1. Comment

The title compound, possessing an intact homodrimane skeleton, is an amide derivative of (+)-sclareolide, which was synthesized from natural sclareol (Barrero *et al.*, 2004). The commercially available diterpene (-)-sclareol or the lactone derivative (+)-sclareolide make an ideal starting point for some biologically important natural products (Mohamad *et al.*, 2005). Furthermore, the enanti­ometrically pure sclareolide provided the perfect tool to validate the absolute stereochemistry of certain chinensine family members, whose stereochemistry had been tentatively assigned based on comparisons to other biogenetically close compounds, such as coronarin E (Margaros & Vassilikogiannakis, 2007; Sy & Brown, 1997). Herein, we report on the first synthesis and crystal structure of the title compound.

The molecular structure of the title compound is shown in Fig. 1. The molecule is composed of three main rings (A, B and C). The six-membered rings, A (C13/C14/C17—C20) and B (C9—C14), are *trans*-fused and have chair conformations. Bond angles to the aliphatic rings and to the aromatic ring C (C1—C6) are in the range of 114.40 (16) to 129.65 (16)° and 117.91 (19) to 122.2 (2)°, respectively. The methyl group at C15 and the side chain at C8 are attached in ideal equatorial positions. There is an intra­molecular N—H···O hydrogen bond forming an S(7) ring motif (Table 1).

In the crystal, O—H···O hydrogen bonds link the molecules into zigzag chains propagating along the *b* axis direction (Table 1 and Fig. 2). The chains are linked via C—H···π inter­actions forming a three-dimensional structure (Table 1).

### S2. Synthesis and crystallization

A solution of DIBAL-H (1.5 M in toluene, 2.58 ml, 3.87 mmol) was added to a cooled (273 K) solution of *o*-methyl­anilines (0.688 g, 4.0 mmol) in THF (1.7 ml) under nitro­gen. The mixture was allowed to warm up and stirred at rt for 2 h. The concentration of the prepared DIBAL-H-o-CH_3_C_6_H_4_NH_2_ complex was *ca* 0.88 M, and was used directly for amino­lysis. To a solution of (+)-sclareolide (0.168 g, 0.67 mmol) in THF (2.5 ml) was added, under nitro­gen at rt, the DIBAL-H-*p*-C_6_H_4_NH_2_ complexe (3.8 ml, 3.35 mmol). After stirring at rt for 2 h, the reaction was cooled to 273 K, and then quenched with H_2_O (1.5 ml) and a 1 M aqueous solution of KHSO_4_ (4 ml). The resulting mixture was extracted with CH_2_Cl_2_ (3 × 10 ml). The combined organic layers were washed with brine, dried over Na_2_SO_4_ and concentrated. The residue was purified by flash chromatography (200-300 m ilicon) with PE/EtOAc = 6:1 as eluant to give the title compound (215 mg, yield 90 %) as a white solid (Margaros & Georgios, 2007). Colourless crystals were obtained by slow evaporation of a solution in CH_2_Cl_2_.

### S2.1. Refinement

Crystal data, data collection and structure refinement details are summarized in Table 2. All the H atoms could be located in difference Fourier maps. In the final cycles of refinement they were included in calculated positions and refined as riding atoms: O—H = 0.82 Å, N—H = 0.86 Å and C—H = 0.93-0.97 Å with U_iso_(H) = 1.5*U*_eq_(C) for OH and methyl H atoms and 1.2U_eq_(N,C) for other H atoms. The absolute configuration of the title compound is based on that of the starting reagent (+)-sclareolide.

### Crystal data

**Table:**

C_23_H_35_NO_2_	*F*(000) = 392
*M**_r_* = 357.52	*D*_x_ = 1.126 Mg m^−^^3^
Monoclinic, *P*2_1_	Mo *K*α radiation, λ = 0.71073 Å
Hall symbol: P 2yb	Cell parameters from 2068 reflections
*a* = 6.3001 (5) Å	θ = 3.1–23.4°
*b* = 13.2663 (10) Å	µ = 0.07 mm^−^^1^
*c* = 12.7082 (10) Å	*T* = 296 K
β = 96.983 (2)°	Block, colorless
*V* = 1054.26 (14) Å^3^	0.22 × 0.20 × 0.18 mm
*Z* = 2	

### Data collection

**Table:**

Bruker SMART APEX CCD diffractometer	3714 independent reflections
Radiation source: fine-focus sealed tube	3121 reflections with *I* > 2σ(*I*)
Graphite monochromator	*R*_int_ = 0.020
phi and ω scans	θ_max_ = 25.4°, θ_min_ = 1.6°
Absorption correction: multi-scan (*SADABS*; Bruker, 2002)	*h* = −7→7
*T*_min_ = 0.985, *T*_max_ = 0.987	*k* = −15→14
3714 measured reflections	*l* = 0→15

### Refinement

**Table:**

Refinement on *F*^2^	Primary atom site location: structure-invariant direct methods
Least-squares matrix: full	Secondary atom site location: difference Fourier map
*R*[*F*^2^ > 2σ(*F*^2^)] = 0.040	Hydrogen site location: inferred from neighbouring sites
*wR*(*F*^2^) = 0.095	H-atom parameters constrained
*S* = 1.06	*w* = 1/[σ^2^(*F*_o_^2^) + (0.0452*P*)^2^ + 0.0489*P*] where *P* = (*F*_o_^2^ + 2*F*_c_^2^)/3
3714 reflections	(Δ/σ)_max_ < 0.001
242 parameters	Δρ_max_ = 0.11 e Å^−^^3^
1 restraint	Δρ_min_ = −0.13 e Å^−^^3^

### Special details

**Table:**

Geometry. All e.s.d.'s (except the e.s.d. in the dihedral angle between two l.s. planes) are estimated using the full covariance matrix. The cell e.s.d.'s are taken into account individually in the estimation of e.s.d.'s in distances, angles and torsion angles; correlations between e.s.d.'s in cell parameters are only used when they are defined by crystal symmetry. An approximate (isotropic) treatment of cell e.s.d.'s is used for estimating e.s.d.'s involving l.s. planes.
Refinement. Refinement of *F*^2^ against ALL reflections. The weighted *R*-factor *wR* and goodness of fit *S* are based on *F*^2^, conventional *R*-factors *R* are based on *F*, with *F* set to zero for negative *F*^2^. The threshold expression of *F*^2^ > σ(*F*^2^) is used only for calculating *R*-factors(gt) *etc*. and is not relevant to the choice of reflections for refinement. *R*-factors based on *F*^2^ are statistically about twice as large as those based on *F*, and *R*- factors based on ALL data will be even larger.

### Fractional atomic coordinates and isotropic or equivalent isotropic displacement parameters (Å^2^)

**Table:**

	*x*	*y*	*z*	*U*_iso_*/*U*_eq_	
O1	0.4624 (2)	0.38763 (11)	0.90592 (12)	0.0598 (4)	
H1O	0.5362	0.4362	0.9269	0.090*	
O2	0.3383 (3)	0.06216 (10)	1.00512 (14)	0.0661 (4)	
N1	0.2698 (2)	0.22934 (12)	1.01913 (11)	0.0413 (4)	
H1	0.3134	0.2876	1.0012	0.050*	
C1	0.0998 (3)	0.23103 (14)	1.08233 (13)	0.0373 (4)	
C2	0.0322 (3)	0.32517 (14)	1.11475 (15)	0.0449 (5)	
C3	−0.1453 (4)	0.32833 (19)	1.16972 (18)	0.0601 (6)	
H3	−0.1947	0.3906	1.1900	0.072*	
C4	−0.2502 (4)	0.2428 (2)	1.19504 (17)	0.0647 (6)	
H4	−0.3692	0.2472	1.2315	0.078*	
C5	−0.1783 (4)	0.1515 (2)	1.16623 (18)	0.0608 (6)	
H5	−0.2466	0.0931	1.1848	0.073*	
C6	−0.0049 (3)	0.14439 (16)	1.10965 (17)	0.0503 (5)	
H6	0.0419	0.0815	1.0898	0.060*	
C7	0.3739 (3)	0.15013 (14)	0.98281 (15)	0.0412 (4)	
C8	0.5396 (3)	0.17800 (15)	0.91191 (16)	0.0448 (5)	
H8A	0.6079	0.1167	0.8914	0.054*	
H8B	0.6485	0.2187	0.9523	0.054*	
C9	0.4531 (3)	0.23662 (13)	0.80977 (13)	0.0351 (4)	
H9	0.2994	0.2439	0.8135	0.042*	
C10	0.5404 (3)	0.34563 (14)	0.81224 (16)	0.0461 (5)	
C11	0.4370 (4)	0.40191 (15)	0.71547 (18)	0.0601 (6)	
H11A	0.5034	0.4677	0.7131	0.072*	
H11B	0.2869	0.4123	0.7224	0.072*	
C12	0.4552 (4)	0.34729 (15)	0.61180 (17)	0.0584 (6)	
H12A	0.6046	0.3408	0.6016	0.070*	
H12B	0.3838	0.3862	0.5532	0.070*	
C13	0.3536 (3)	0.24265 (16)	0.61310 (15)	0.0458 (5)	
H13	0.2113	0.2551	0.6342	0.055*	
C14	0.4691 (3)	0.17743 (14)	0.70513 (14)	0.0380 (4)	
C15	0.7835 (4)	0.35592 (19)	0.82392 (19)	0.0667 (7)	
H15A	0.8226	0.4244	0.8415	0.100*	
H15B	0.8349	0.3379	0.7584	0.100*	
H15C	0.8459	0.3120	0.8793	0.100*	
C16	0.7032 (3)	0.15146 (18)	0.69257 (18)	0.0558 (5)	
H16A	0.7818	0.1407	0.7612	0.084*	
H16B	0.7663	0.2061	0.6578	0.084*	
H16C	0.7073	0.0913	0.6508	0.084*	
C17	0.3428 (3)	0.07858 (15)	0.71010 (17)	0.0507 (5)	
H17A	0.4192	0.0347	0.7628	0.061*	
H17B	0.2047	0.0936	0.7329	0.061*	
C18	0.3081 (5)	0.02307 (19)	0.6044 (2)	0.0782 (8)	
H18A	0.4451	0.0016	0.5847	0.094*	
H18B	0.2220	−0.0366	0.6116	0.094*	
C19	0.1974 (4)	0.0897 (2)	0.5183 (2)	0.0848 (9)	
H19A	0.0535	0.1032	0.5343	0.102*	
H19B	0.1858	0.0530	0.4518	0.102*	
C20	0.3085 (4)	0.1906 (2)	0.50341 (17)	0.0658 (6)	
C21	0.5087 (5)	0.1731 (3)	0.4470 (2)	0.0896 (9)	
H21A	0.6003	0.1248	0.4861	0.134*	
H21B	0.5843	0.2356	0.4431	0.134*	
H21C	0.4663	0.1482	0.3767	0.134*	
C22	0.1549 (6)	0.2562 (3)	0.4303 (2)	0.1163 (13)	
H22A	0.1083	0.2197	0.3664	0.174*	
H22B	0.2268	0.3167	0.4131	0.174*	
H22C	0.0333	0.2732	0.4655	0.174*	
C23	0.1454 (4)	0.42079 (16)	1.0915 (2)	0.0669 (7)	
H23A	0.0722	0.4774	1.1175	0.100*	
H23B	0.1467	0.4273	1.0164	0.100*	
H23C	0.2897	0.4188	1.1259	0.100*	

### Atomic displacement parameters (Å^2^)

**Table:**

	*U*^11^	*U*^22^	*U*^33^	*U*^12^	*U*^13^	*U*^23^
O1	0.0856 (11)	0.0399 (8)	0.0583 (9)	−0.0191 (7)	0.0266 (8)	−0.0166 (7)
O2	0.0854 (11)	0.0327 (8)	0.0842 (11)	0.0119 (8)	0.0266 (9)	0.0162 (8)
N1	0.0510 (9)	0.0274 (8)	0.0467 (9)	−0.0027 (7)	0.0110 (7)	0.0035 (7)
C1	0.0414 (9)	0.0384 (10)	0.0314 (9)	−0.0004 (9)	0.0012 (7)	0.0030 (8)
C2	0.0536 (12)	0.0420 (11)	0.0392 (11)	0.0005 (9)	0.0056 (9)	−0.0023 (8)
C3	0.0650 (14)	0.0676 (16)	0.0497 (13)	0.0086 (13)	0.0145 (11)	−0.0088 (11)
C4	0.0582 (13)	0.092 (2)	0.0467 (13)	0.0002 (14)	0.0160 (10)	0.0072 (13)
C5	0.0603 (13)	0.0724 (16)	0.0495 (13)	−0.0172 (12)	0.0059 (11)	0.0188 (12)
C6	0.0605 (13)	0.0429 (11)	0.0473 (12)	−0.0045 (10)	0.0051 (10)	0.0092 (9)
C7	0.0497 (11)	0.0344 (11)	0.0379 (10)	0.0053 (9)	−0.0009 (8)	0.0056 (8)
C8	0.0452 (11)	0.0438 (11)	0.0449 (11)	0.0107 (9)	0.0044 (8)	−0.0001 (9)
C9	0.0365 (9)	0.0314 (9)	0.0383 (10)	0.0025 (8)	0.0075 (7)	0.0001 (8)
C10	0.0594 (12)	0.0352 (11)	0.0459 (12)	−0.0093 (9)	0.0151 (9)	−0.0071 (8)
C11	0.0855 (16)	0.0337 (12)	0.0642 (15)	−0.0013 (11)	0.0215 (12)	0.0091 (10)
C12	0.0787 (15)	0.0495 (13)	0.0489 (13)	0.0011 (11)	0.0152 (11)	0.0159 (10)
C13	0.0433 (10)	0.0532 (12)	0.0415 (11)	0.0001 (9)	0.0079 (8)	0.0025 (9)
C14	0.0395 (10)	0.0366 (10)	0.0397 (10)	−0.0017 (8)	0.0116 (8)	−0.0019 (7)
C15	0.0656 (14)	0.0696 (16)	0.0664 (15)	−0.0320 (12)	0.0143 (11)	−0.0156 (12)
C16	0.0493 (12)	0.0572 (13)	0.0639 (14)	0.0062 (10)	0.0193 (10)	−0.0053 (11)
C17	0.0610 (13)	0.0417 (12)	0.0520 (13)	−0.0102 (10)	0.0172 (10)	−0.0090 (9)
C18	0.104 (2)	0.0623 (17)	0.0738 (19)	−0.0359 (15)	0.0339 (16)	−0.0293 (13)
C19	0.0888 (19)	0.111 (2)	0.0561 (17)	−0.0440 (17)	0.0149 (14)	−0.0369 (16)
C20	0.0702 (14)	0.0887 (18)	0.0385 (12)	−0.0182 (13)	0.0066 (11)	−0.0055 (11)
C21	0.106 (2)	0.117 (2)	0.0529 (16)	−0.0345 (18)	0.0368 (15)	−0.0212 (15)
C22	0.117 (3)	0.166 (4)	0.0575 (17)	−0.009 (2)	−0.0239 (17)	0.0145 (19)
C23	0.0797 (16)	0.0373 (12)	0.0870 (18)	−0.0010 (11)	0.0236 (14)	−0.0140 (11)

### Geometric parameters (Å, º)

**Table:**

O1—C10	1.453 (2)	C13—C20	1.551 (3)
O1—H1O	0.8200	C13—C14	1.562 (3)
O2—C7	1.228 (2)	C13—H13	0.9800
N1—C7	1.349 (2)	C14—C17	1.539 (3)
N1—C1	1.415 (2)	C14—C16	1.541 (3)
N1—H1	0.8600	C15—H15A	0.9600
C1—C6	1.390 (3)	C15—H15B	0.9600
C1—C2	1.398 (3)	C15—H15C	0.9600
C2—C3	1.390 (3)	C16—H16A	0.9600
C2—C23	1.502 (3)	C16—H16B	0.9600
C3—C4	1.371 (3)	C16—H16C	0.9600
C3—H3	0.9300	C17—C18	1.524 (3)
C4—C5	1.358 (4)	C17—H17A	0.9700
C4—H4	0.9300	C17—H17B	0.9700
C5—C6	1.382 (3)	C18—C19	1.510 (4)
C5—H5	0.9300	C18—H18A	0.9700
C6—H6	0.9300	C18—H18B	0.9700
C7—C8	1.506 (3)	C19—C20	1.533 (4)
C8—C9	1.554 (3)	C19—H19A	0.9700
C8—H8A	0.9700	C19—H19B	0.9700
C8—H8B	0.9700	C20—C22	1.529 (4)
C9—C10	1.546 (3)	C20—C21	1.542 (4)
C9—C14	1.558 (2)	C21—H21A	0.9600
C9—H9	0.9800	C21—H21B	0.9600
C10—C11	1.516 (3)	C21—H21C	0.9600
C10—C15	1.527 (3)	C22—H22A	0.9600
C11—C12	1.520 (3)	C22—H22B	0.9600
C11—H11A	0.9700	C22—H22C	0.9600
C11—H11B	0.9700	C23—H23A	0.9600
C12—C13	1.530 (3)	C23—H23B	0.9600
C12—H12A	0.9700	C23—H23C	0.9600
C12—H12B	0.9700		
			
C10—O1—H1O	109.5	C17—C14—C16	108.65 (16)
C7—N1—C1	129.73 (16)	C17—C14—C9	107.87 (14)
C7—N1—H1	115.1	C16—C14—C9	111.29 (15)
C1—N1—H1	115.1	C17—C14—C13	107.85 (15)
C6—C1—C2	119.63 (17)	C16—C14—C13	114.27 (15)
C6—C1—N1	122.93 (18)	C9—C14—C13	106.69 (14)
C2—C1—N1	117.40 (16)	C10—C15—H15A	109.5
C3—C2—C1	117.90 (19)	C10—C15—H15B	109.5
C3—C2—C23	120.25 (19)	H15A—C15—H15B	109.5
C1—C2—C23	121.85 (18)	C10—C15—H15C	109.5
C4—C3—C2	122.2 (2)	H15A—C15—H15C	109.5
C4—C3—H3	118.9	H15B—C15—H15C	109.5
C2—C3—H3	118.9	C14—C16—H16A	109.5
C5—C4—C3	119.3 (2)	C14—C16—H16B	109.5
C5—C4—H4	120.4	H16A—C16—H16B	109.5
C3—C4—H4	120.4	C14—C16—H16C	109.5
C4—C5—C6	120.8 (2)	H16A—C16—H16C	109.5
C4—C5—H5	119.6	H16B—C16—H16C	109.5
C6—C5—H5	119.6	C18—C17—C14	113.26 (17)
C5—C6—C1	120.2 (2)	C18—C17—H17A	108.9
C5—C6—H6	119.9	C14—C17—H17A	108.9
C1—C6—H6	119.9	C18—C17—H17B	108.9
O2—C7—N1	123.46 (17)	C14—C17—H17B	108.9
O2—C7—C8	122.06 (17)	H17A—C17—H17B	107.7
N1—C7—C8	114.48 (16)	C19—C18—C17	111.1 (2)
C7—C8—C9	115.10 (15)	C19—C18—H18A	109.4
C7—C8—H8A	108.5	C17—C18—H18A	109.4
C9—C8—H8A	108.5	C19—C18—H18B	109.4
C7—C8—H8B	108.5	C17—C18—H18B	109.4
C9—C8—H8B	108.5	H18A—C18—H18B	108.0
H8A—C8—H8B	107.5	C18—C19—C20	115.0 (2)
C10—C9—C8	111.30 (15)	C18—C19—H19A	108.5
C10—C9—C14	115.40 (14)	C20—C19—H19A	108.5
C8—C9—C14	114.09 (15)	C18—C19—H19B	108.5
C10—C9—H9	104.9	C20—C19—H19B	108.5
C8—C9—H9	104.9	H19A—C19—H19B	107.5
C14—C9—H9	104.9	C22—C20—C19	107.9 (2)
O1—C10—C11	108.73 (17)	C22—C20—C21	107.2 (2)
O1—C10—C15	108.72 (17)	C19—C20—C21	109.7 (2)
C11—C10—C15	111.21 (18)	C22—C20—C13	109.0 (2)
O1—C10—C9	102.74 (14)	C19—C20—C13	108.33 (18)
C11—C10—C9	109.11 (16)	C21—C20—C13	114.47 (19)
C15—C10—C9	115.82 (17)	C20—C21—H21A	109.5
C10—C11—C12	113.45 (17)	C20—C21—H21B	109.5
C10—C11—H11A	108.9	H21A—C21—H21B	109.5
C12—C11—H11A	108.9	C20—C21—H21C	109.5
C10—C11—H11B	108.9	H21A—C21—H21C	109.5
C12—C11—H11B	108.9	H21B—C21—H21C	109.5
H11A—C11—H11B	107.7	C20—C22—H22A	109.5
C11—C12—C13	110.33 (17)	C20—C22—H22B	109.5
C11—C12—H12A	109.6	H22A—C22—H22B	109.5
C13—C12—H12A	109.6	C20—C22—H22C	109.5
C11—C12—H12B	109.6	H22A—C22—H22C	109.5
C13—C12—H12B	109.6	H22B—C22—H22C	109.5
H12A—C12—H12B	108.1	C2—C23—H23A	109.5
C12—C13—C20	115.19 (17)	C2—C23—H23B	109.5
C12—C13—C14	110.69 (16)	H23A—C23—H23B	109.5
C20—C13—C14	116.36 (18)	C2—C23—H23C	109.5
C12—C13—H13	104.3	H23A—C23—H23C	109.5
C20—C13—H13	104.3	H23B—C23—H23C	109.5
C14—C13—H13	104.3		
			
C7—N1—C1—C6	−6.7 (3)	C11—C12—C13—C20	164.77 (19)
C7—N1—C1—C2	175.54 (18)	C11—C12—C13—C14	−60.6 (2)
C6—C1—C2—C3	−2.8 (3)	C10—C9—C14—C17	−170.84 (16)
N1—C1—C2—C3	175.04 (18)	C8—C9—C14—C17	58.41 (19)
C6—C1—C2—C23	177.3 (2)	C10—C9—C14—C16	70.1 (2)
N1—C1—C2—C23	−4.9 (3)	C8—C9—C14—C16	−60.7 (2)
C1—C2—C3—C4	1.8 (3)	C10—C9—C14—C13	−55.19 (19)
C23—C2—C3—C4	−178.3 (2)	C8—C9—C14—C13	174.06 (14)
C2—C3—C4—C5	0.4 (3)	C12—C13—C14—C17	173.35 (16)
C3—C4—C5—C6	−1.7 (3)	C20—C13—C14—C17	−52.6 (2)
C4—C5—C6—C1	0.6 (3)	C12—C13—C14—C16	−65.7 (2)
C2—C1—C6—C5	1.7 (3)	C20—C13—C14—C16	68.3 (2)
N1—C1—C6—C5	−176.07 (18)	C12—C13—C14—C9	57.69 (19)
C1—N1—C7—O2	−3.4 (3)	C20—C13—C14—C9	−168.28 (15)
C1—N1—C7—C8	176.65 (17)	C16—C14—C17—C18	−70.6 (2)
O2—C7—C8—C9	120.4 (2)	C9—C14—C17—C18	168.6 (2)
N1—C7—C8—C9	−59.7 (2)	C13—C14—C17—C18	53.7 (2)
C7—C8—C9—C10	113.33 (18)	C14—C17—C18—C19	−56.3 (3)
C7—C8—C9—C14	−113.93 (17)	C17—C18—C19—C20	55.1 (3)
C8—C9—C10—O1	−60.06 (18)	C18—C19—C20—C22	−168.8 (2)
C14—C9—C10—O1	167.86 (14)	C18—C19—C20—C21	74.7 (3)
C8—C9—C10—C11	−175.33 (16)	C18—C19—C20—C13	−51.0 (3)
C14—C9—C10—C11	52.6 (2)	C12—C13—C20—C22	−60.1 (3)
C8—C9—C10—C15	58.3 (2)	C14—C13—C20—C22	168.0 (2)
C14—C9—C10—C15	−73.8 (2)	C12—C13—C20—C19	−177.2 (2)
O1—C10—C11—C12	−163.68 (17)	C14—C13—C20—C19	50.8 (3)
C15—C10—C11—C12	76.6 (2)	C12—C13—C20—C21	60.0 (3)
C9—C10—C11—C12	−52.3 (2)	C14—C13—C20—C21	−72.0 (3)
C10—C11—C12—C13	58.1 (3)		

### Hydrogen-bond geometry (Å, º)

Cg is the centroid of benzene ring C1–C6.

**Table:**

*D*—H···*A*	*D*—H	H···*A*	*D*···*A*	*D*—H···*A*
N1—H1···O1	0.86	2.09	2.894 (2)	155
O1—H1*O*···O2^i^	0.82	2.00	2.8054 (19)	168
C8—H8*B*···*Cg*^ii^	0.97	2.79	3.632 (2)	146
C22—H22*A*···*Cg*^iii^	0.96	2.98	3.808 (3)	145

Symmetry codes: (i) −*x*+1, *y*+1/2, −*z*+2; (ii) *x*+1, *y*, *z*; (iii) *x*, *y*, *z*−1.

## References

[bb1] Barrero, A. F., Alvarez–Manzaneda, E. J., Chahboun, R. & Arteaga, A. F. (2004). *Synth. Commun.* **34**, 3631–3643.

[bb2] Bernardinelli, G. & Giersch, W. (1985). *Acta Cryst.* C**41**, 746–749.

[bb3] Bruker (2002). *SMART*, *SAINT* and *SADABS*. Bruker AXS Inc., Madison, Wisconsin, USA.

[bb4] Huang, P.-Q., Zheng, X. & Deng, X.-M. (2001). *Tetrahedron Lett.* **42**, 9039–9041.

[bb5] Margaros, I. & Vassilikogiannakis, G. (2007). *J. Org. Chem.* **72**, 4826–4831. 10.1021/jo070527v 17530899

[bb6] Mohamad, H., Lajis, N. H., Abas, F., Ali, A. M., Sukari, M. A., Kikuzaki, H. & Nakatani, N. (2005). *J. Nat. Prod.* **68**, 285–288. 10.1021/np040098l 15730265

[bb7] Sheldrick, G. M. (2008). *Acta Cryst.* A**64**, 112–122. 10.1107/S0108767307043930 18156677

[bb8] Shi, X.-W., Li, S.-K., Li, D.-D. & Lu, Q.-Q. (2015). *Acta Cryst.* E**71**, o710–o711. 10.1107/S2056989015016370 PMC4647384 26594436

[bb9] Sy, L.-K. & Brown, G. D. (1997). *J. Nat. Prod.* **60**, 904–908.

