# Supplementary figures and images for: Crystal structure of 2-[(1R,2R,4aS,8aS)-2-hy­droxy-2,5,5,8a-tetra­methyl­deca­hydro­naphthalen-1-yl]-N-(o-tol­yl)acetamide
Source: Acta Crystallogr E Crystallogr Commun. 2015 Sep 26;71(Pt 10):o788–9. doi: 10.1107/S2056989015017600 (PMC4647428; doi:10.1107/S2056989015017600)

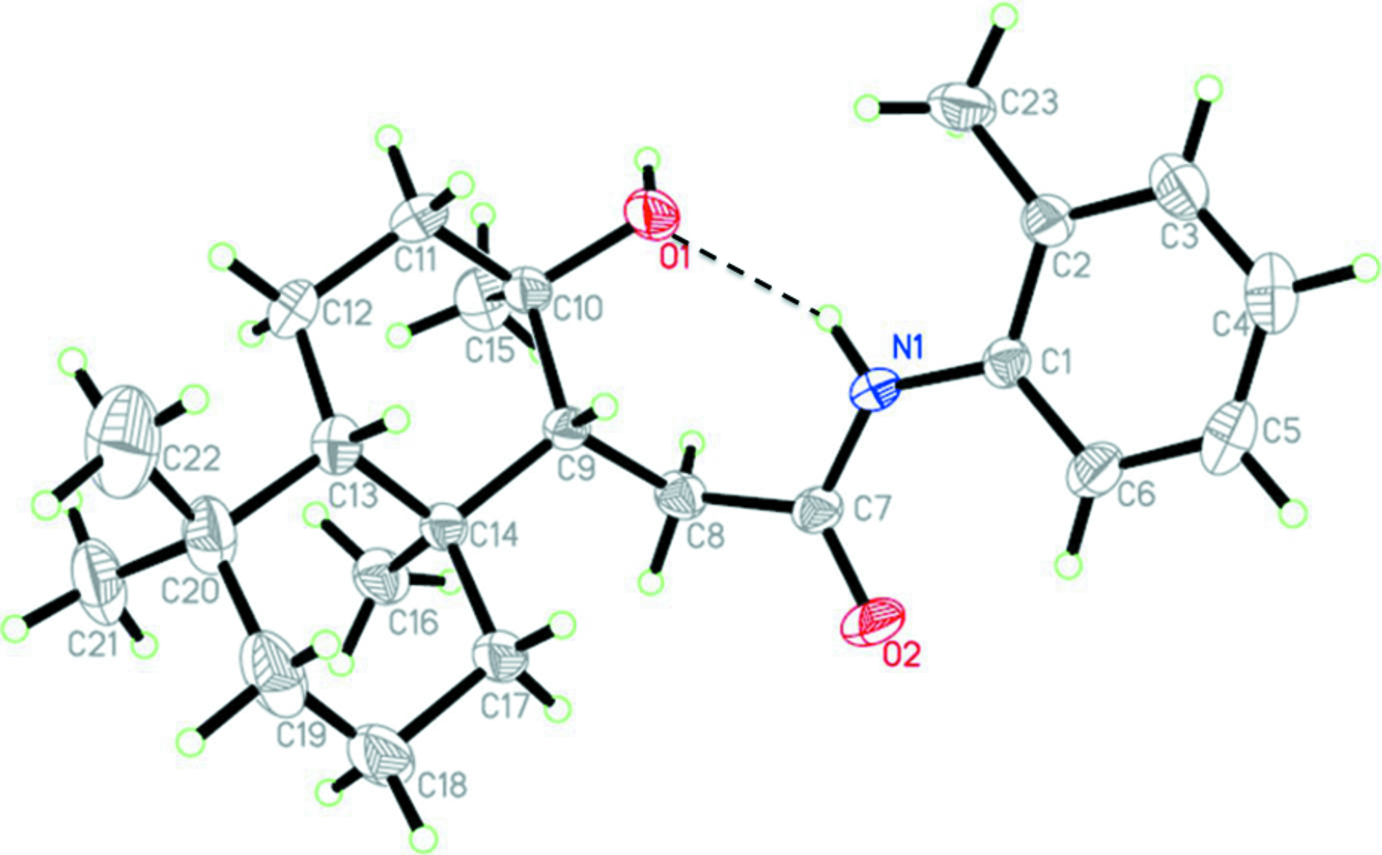

Supplement: Supplementary file 4 [file e-71-0o788-fig1.tif]

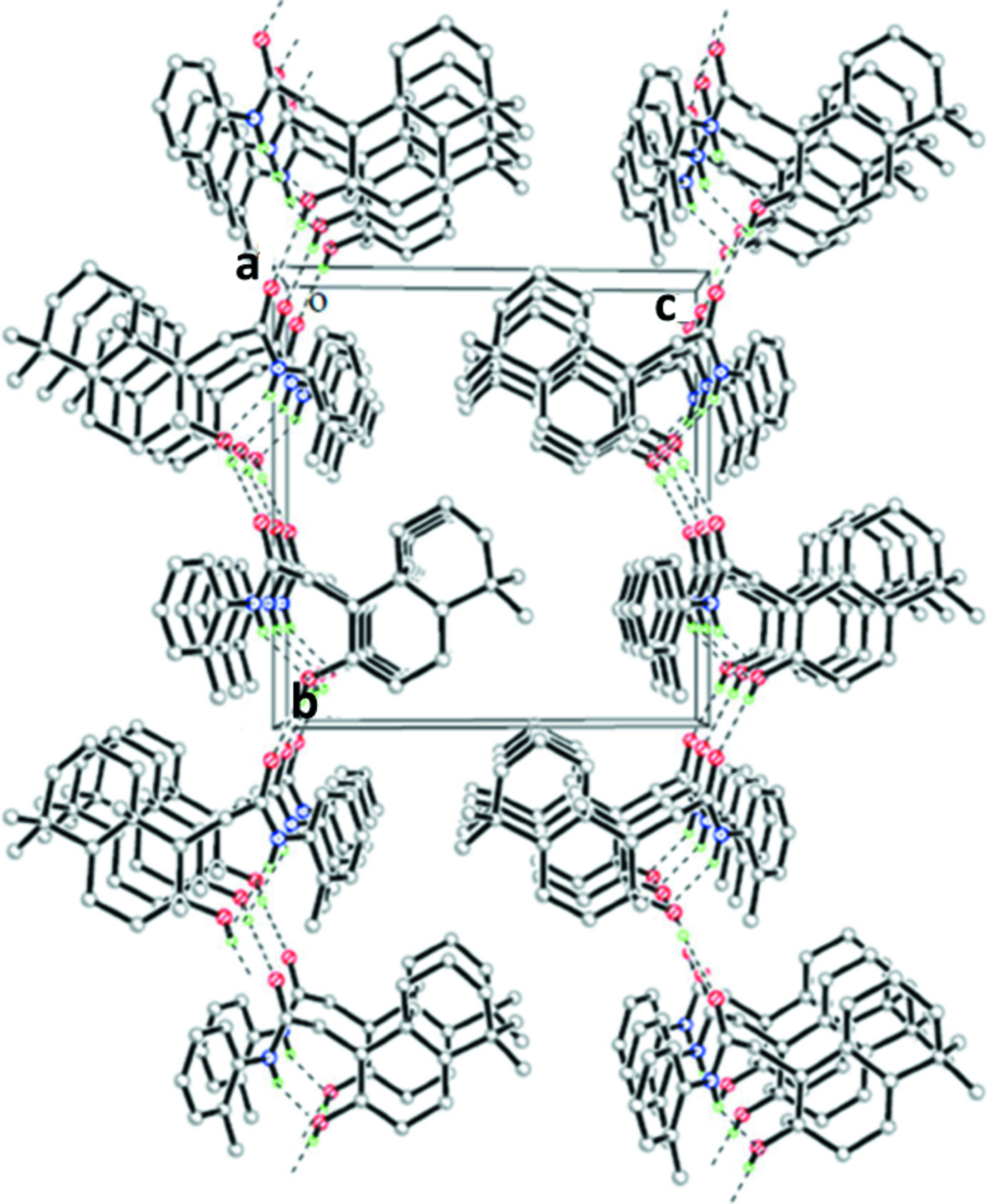

Supplement: Supplementary file 5 [file e-71-0o788-fig2.tif]
